# RIG-I Self-Oligomerization Is Either Dispensable or Very Transient for Signal Transduction

**DOI:** 10.1371/journal.pone.0108770

**Published:** 2014-09-26

**Authors:** Jade Louber, Eva Kowalinski, Louis-Marie Bloyet, Joanna Brunel, Stephen Cusack, Denis Gerlier

**Affiliations:** 1 Centre International de Recherche en Infectiologie, INSERM, U1111, CNRS, UMR5308, Université Lyon 1, ENS Lyon, Lyon, France; 2 European Molecular Biology Laboratory, Grenoble Outstation, Grenoble Cedex 9, France; 3 Unit of Virus Host-Cell Interactions, UJF-EMBL-CNRS, UMI 3265, Grenoble Cedex 9, France; INRA, France

## Abstract

Effective host defence against viruses depends on the rapid triggering of innate immunity through the induction of a type I interferon (IFN) response. To this end, microbe-associated molecular patterns are detected by dedicated receptors. Among them, the RIG-I-like receptors RIG-I and MDA5 activate IFN gene expression upon sensing viral RNA in the cytoplasm. While MDA5 forms long filaments *in vitro* upon activation, RIG-I is believed to oligomerize after RNA binding in order to transduce a signal. Here, we show that *in vitro* binding of synthetic RNA mimicking that of *Mononegavirales* (Ebola, rabies and measles viruses) leader sequences to purified RIG-I does not induce RIG-I oligomerization. Furthermore, in cells devoid of endogenous functional RIG-I-like receptors, after activation of exogenous Flag-RIG-I by a 62-mer-5′ppp-dsRNA or by polyinosinic:polycytidylic acid, a dsRNA analogue, or by measles virus infection, anti-Flag immunoprecipitation and specific elution with Flag peptide indicated a monomeric form of RIG-I. Accordingly, when using the *Gaussia* Luciferase-Based Protein Complementation Assay (PCA), a more sensitive *in cellula* assay, no RIG-I oligomerization could be detected upon RNA stimulation. Altogether our data indicate that the need for self-oligomerization of RIG-I for signal transduction is either dispensable or very transient.

## Introduction

In vertebrates, the first step of innate immunity is the detection of microbe-associated molecular patterns (MAMPs) by specific pattern-recognition receptors (PRRs) [1], [2]. RIG-I (retinoic acid-inducible gene I) belongs to the cytoplasmic RIG-I-like receptors (RLRs) together with MDA5 (melanoma differentiation-associated protein 5) and LGP2 (laboratory of genetics and physiology 2). In response to infection by RNA viruses, RIG-I activates type-1 interferon (IFN) genes [1], [3], [4], [5], [6]. RIG-I consists of two amino-terminal caspase activation and recruitment domains (CARDs) that are essential for signal transduction, a central helicase and a C-terminal domain both of which bind an agonist RNA. The mechanism of RIG-I activation has been widely studied over the past few years. RIG-I preferentially recognizes 5′-triphosphorylated (5′ppp) blunt ended double-stranded RNA, but it can also bind to long double-stranded RNA (dsRNA) without 5′ppp [7], [8], [9], [10]. The recognition of an agonist RNA triggers a conformational change, allowing RIG-I to become active thanks to the release of the CARD domains. The free CARDs are then accessible for poly-ubiquitination and recruitment of the adaptor mitochondrial antiviral signal (MAVS) protein [1], [11], [12], [13].

The precise mechanisms of RIG-I activation are still not fully understood. It has been proposed that RIG-I-mediated activation relies on RIG-I oligomerization via dimerization of RIG-I C-terminal domain (CTD), multiple oligomerization sites within RIG-I, and/or RNA-mediated oligomerization [7], [10], [14], [15], [16], [17], [18], [19], [20], [21]. In the present study, we question the necessity of RIG-I self-oligomerization for signal induction. RIG-I oligomerization, induced by synthetic cognate RNA able to activate RIG-I and as well as activation by measles virus (MeV), was analysed by co-immunoprecipitation and a sensitive protein complementation assay. In the absence of convincing evidence of self-oligomerization our data support monomeric RIG-I as being the minimal signal transduction unit.

## Materials and Methods

### Cells and virus

Huh7.5 [22], Vero [23] and 293T [24] cells were maintained in Dulbecco’s modified Eagle’s medium (DMEM Gibco, Invitrogen) supplemented with 10% foetal calf serum (Gibco), 10 mM HEPES, 2 mM L-glutamine, 10 µg/ml gentamycine and 1% non-essential amino acids for Huh7.5 cells at 37°C and 5% CO_2_.

Moraten-eGFP measles virus was recovered by reverse genetics as described by Radecke et al. [25]. The helper cell line 293-3-46 stably expressing T7 polymerase, MeV N and P proteins [25] was transfected using the ProFection kit (Promega) with plasmids coding for MeV genome with an additional eGFP gene and MeV-L protein (pEMCLa). Three days after transfection, cells were overlaid on Vero cells. Upon appearance, isolated syncytia were picked and individually propagated on Vero cells. Virus stock was produced after a second passage at multiplicity of infection (MOI) 0.03 on Vero cells. Virus was checked for lack of mycoplasma contamination, sequence accuracy and infectivity (virus titration).

### Plasmids

Wild-type human RIG-I and RIG-I^ko^ (T55I, Q229A, T697A, E702A, K888A, K907A) cDNA were subcloned into pEF-BOS expression vector using PCR amplification of cDNA fragments and in vitro recombination (InFusion, Clontech). HA, Cl25 (Ghannam et al., 2008) and Flag tag coding sequences were fused to RIG-I cDNA during the PCR amplification step. RIG-I insert constructs were entirely verified by sequencing (Eurofins).

The two original expression vectors used for *Gaussia* Luciferase-Based Protein Complementation Assay (PCA) (Cassonnet et al., 2011), were modified into pCI-glu1 and pCI-glu2 to eliminate the Gateway insert without changing the flanking vector sequence in order to preserve the linker bridging glu domains and inserts. HA-RIG-I and Cl25-RIG-I fragments were subcloned upstream or downstream of gaussia glu1 and/or glu2 domains by InFusion recombination of PCR-amplified fragments. Gcn4 sequence [26] was subcloned upstream or downstream of RIG-I coding sequence by InFusion recombination of PCR-amplified fragments. All plasmids were verified by sequencing of every subcloned PCR fragment.

### Antibodies and reagents

For immunoblotting the following primary antibodies were used: anti-Flag (1∶1,000; M2, Sigma), Cl25 anti-MeV N (1∶1,000) [27], anti-HA (1∶1,000; Clone HA-7, Sigma), 49.21 anti-MeV P (1∶2,000) [28] anti-GAPDH (1∶2000; Millipore) murine monoclonal antibodies and anti-human RIG-I (1∶10,000) rabbit polyclonal antibodies [29].

For DNA plasmid transfection, JetPRIME reagent (Polyplus transfection) was used in 293T cells and Transit-LT1 reagent (Mirus) was used in Huh7.5 cells. RNA transfection was performed with Oligofectamine reagent (Invitrogen). Poly(I:C) was purchased from Amersham Biosciences.

Rabies leader 5′ppp-RNA (GGACGCUUAACAACAAAACCAGAGAAGAAAAAGACAGCGUCAAUUGCAAACGAAAAAUGUGC), measles leader 5′ppp-RNA (GGACCAAACAAAGUUGGGUAAGGAUAGAUCAAUCAAUGAUCAUAUUCUAGUACACUUGAAUUC) and Ebola leader 5′ppp-RNA (GGACACACAAAAAGAAAGAAAAGUUUUUUATACUUUUUGUGUGCGAAUAACUAUG) were *in vitro* T7 transcribed and purified by excising the band after denaturing urea-PAGE [30].

The 62-mer-5′ppp-dsRNA was obtained by annealing two T7 transcribed and purified complementary 62-mer-5′ppp-ssRNA (GGUCCUGUCUGUUGUCGGUCUCGUUUGUUGCGUGUCCGUGUUCGCCUUGGUUCCCCGGUGCC) and (GGCACCGGGGAACCAAGGCGAACACGGACACGCAACAAACGAGACCGACAACAGACAGGACC). Both 62-mer-5′ppp-ssRNA were made from only three nucleotides to avoid secondary structure and preclude T7 polymerase re-initialization on and copy of the nascent RNA [31].

### SEC MALLS

Purified recombinant human RIG-I was prepared as previously described [30] and mixed with equimolar amounts of RNA. Experiments were performed in 20 mM HEPES, pH 7.5, 100 mM NaCl, 2.5 mM MgCl2, 5 mM β-mercaptoethanol with an S200(10/300) column, connected to a MALLS detector (DAWN-EOS Wyatt technology) and a refractive index detector (RI2000b Schambeck). Data were analysed with the ASTRA V software [32].

### SAXS

The experiments were carried out at the beamline ID14-2 of the European Synchrotron Radiation Facility (ESRF, Grenoble, France). Scattering data was collected for different protein concentrations and from the merged curves the radius of gyration (Rg) was determined from the Guinier plot. Next, the maximal distance in the size distribution function was adjusted, so the calculated Rg from the fit would be in agreement with the experimental value.

### Luciferase assay

Cells were seeded into 96-well plates and, 18 h later, transfected with 50 ng DNA of IFN-β luciferase, 17 ng DNA of renilla luciferase and 33 ng DNA of RIG-I plasmid. One day after DNA transfection, cells were transfected with Poly(I:C), synthetic RNA or infected with Moraten-gfp virus at MOI 1. The following day, the luciferase assay was performed using the Dual-Glo system from Promega. Firefly luciferase values were normalized to renilla luciferase to measure transfection efficiency.

For *Gaussia* Luciferase-Based Complementation Assay (PCA) [33], cells were seeded into 96-well plates and, 8 h later, transfected with 100 ng RIG-I-glu1 construct and 100 ng RIG-I-glu2 construct. Twenty four hours after DNA transfection, cells were transfected or not with Poly(I:C). Eighteen hours later, the luciferase assay was performed using the Renilla Luciferase Assay System (Promega). Protein-protein interaction levels were expressed in normalized luminescence ratio (NLR) according to the following formula:
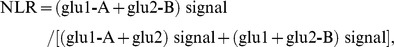
where glu1-A and glu2-B are the chimeric proteins, and glu1 and glu2 the empty vector coding only for the glu fragment.

### Immunoprecipitation and immunoblot analysis

For immunoblot analysis, transfected or infected cells were suspended in lysis buffer, either PLB buffer (10 mM HEPES pH 7.4, 100 mM NaCl, 5 mM MgCl_2_, 0.05% NP-40, 25 mM EDTA) or NP-40 buffer (50 mM Tris HCl pH 7.4, 150 mM NaCl, 0.1% NP-40, 1 mM EDTA) both complemented with Complete (Roche) protease inhibitor cocktail for 20 minutes on ice. The proteins were then separated from cell debris by centrifugation at 7,000×g for 10 minutes. Proteins were denatured by addition of Laemmli 1X buffer and heating at 100°C for 3 minutes before analysis by SDS-PAGE and immunoblotting.

For co-immunoprecipitation analysis, lysates were incubated from 2 h to 16 h at 4°C on a rotating wheel with anti-Flag (M2) beads (Sigma). Beads were washed four times with lysis buffer and proteins were eluted by addition of 22.5 µg 3xFlag Peptides (Sigma). Lysates were then analysed by SDS-PAGE and immunoblotting.

### RNA extraction and amplification

RNA immunoprecipitated with RIG-I was purified by Trizol/chloroform extraction, then amplified by stem-loop RT-PCR as set up for miRNA detection [34] using the Reverse Transcriptase Superscript™ II from Invitrogen and the Taq Polymerase from New England Biolabs. Stem-loop primer used for retrotranscription of measles leader RNAs was GCGACGTTCCGTTGCGATCAGCGTACGCTGATCGCAACGGAACGTCGCcatagt and PCR primers were accaaacaaagttgggtaagg and GCTGCTACTCGGCTGATCTCAC.

## Results

### Oligomer state of RIG-I:RNA complexes formed *in* *vitro*

**Figure 1:**
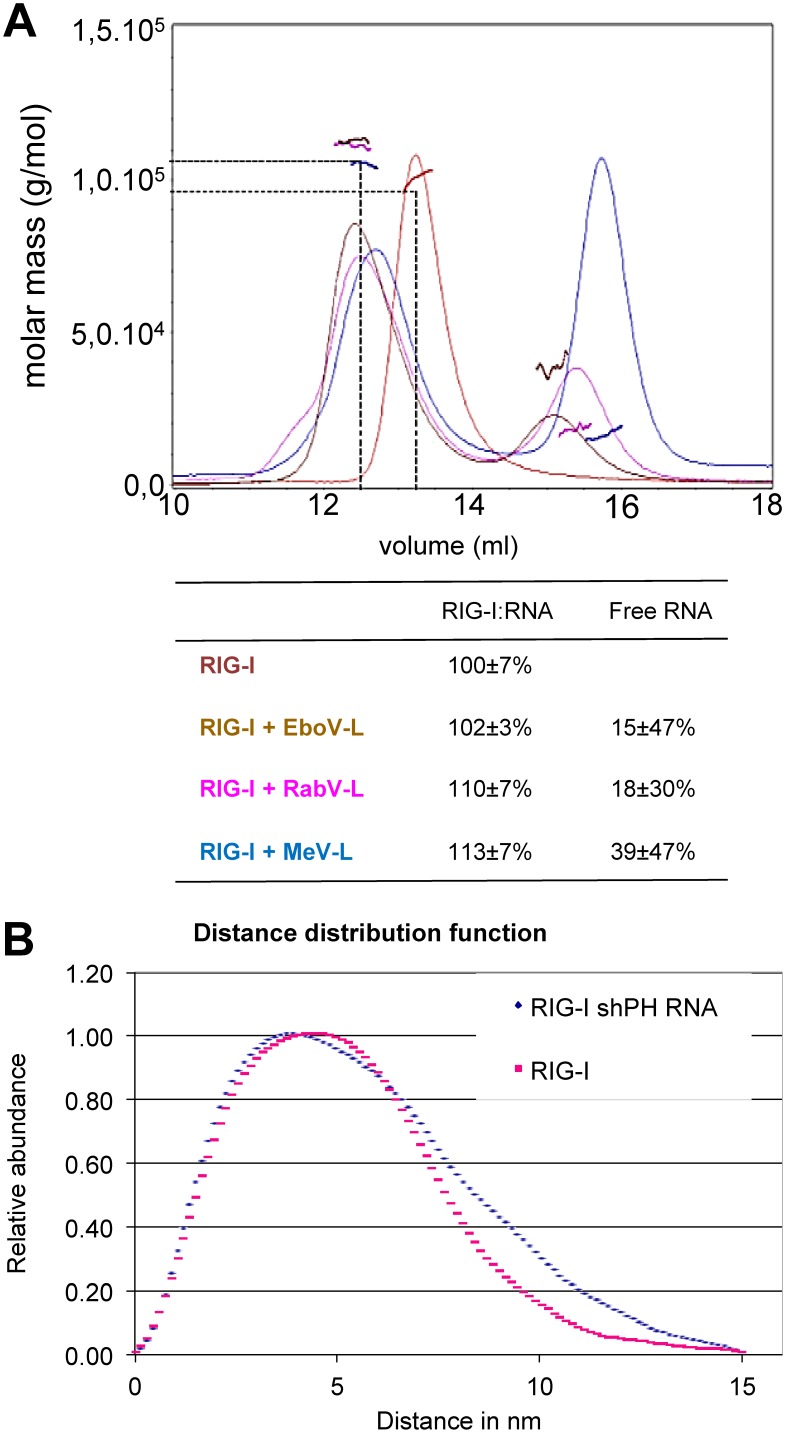
Oligomeric state of RIG-I:RNA complexes produced *in vitro* as determined by SEC MALLS (A) and SAXS (B). (A) 37 mM RIG-I and RIG-I:RNA complexes formed by incubation with 40 mM of RNA and 2 mM ATP analogue were analysed by size-exclusion chromatography on a S200 column coupled to multi-angle laser light scattering. Free RIG-I as well as the RIG-I:RNA complex elutes as monomers or 1∶1 complexes, respectively, with indicated apparent molecular weights. Theoretical values are 106 kDa for RIG-I and 11.8 kDa for the RNA. (B) Scattering data was collected for different protein concentrations of RIG-I or RIG-I:RNA complex and from the merged curves. shPH RNA is an influenza virus derived short pan-handle RNA. The radius of gyration (R_g_) was determined from the Guinier plot. P(R) functions of the scattering curves that were fitted to attain the experimental R_g_ show both a maximal intramolecular distance of 150 Å.

The ability of human RIG-I (hRIG-I) protein to bind different 5′ppp RNA *in vitro* was tested by Multi-Angle Laser Light Scattering coupled to Size Exclusion Column (SEC-MALLS) analysis. Purified recombinant hRIG-I was able to bind to synthetic copies of the leader RNAs from three different *Mononegavirales* families: Ebola (*Filoviridae)*, rabies (*Rhabdoviridae*) and measles (*Paramyxoviridae*) viruses (Figure 1A). The incubation of hRIG-I with each synthetic leader RNA induced a shift to a lower elution volume which indicates a larger, more elongated or less globular particle. This observation points to a conformational change of the protein molecule: either the addition of the RNA moiety to one end of the protein elongates the whole complex, or large parts of bound RNA are flexible and floppy. However, when looking at the apparent molecular masses from MALLS, none of the complexes showed a significant mass shift. RIG-I alone appeared with a mass of 100 kDa, slightly smaller than the calculated mass of 106 kDa, but within the error range. RIG-I associated with the leader RNA of Ebola, rabies or measles virus appeared with a mass of 102 kDa, 110 kDa and 113 kDa, respectively. Moreover, the resulting complex was monodispersed (one peak, flat MALLS signal). It can be concluded that RIG-I binds to each of these RNAs and forms a homogeneous complex with a 1∶1 stoichiometry. When examined by Small Angle X-Ray Scattering (SAXS) the radius of gyration R_g_ for RIG-I without RNA appears to be 38.5±0.27 Å and with a short panhandle RNA of influenza virus to be slightly larger 42.2±0.21 Å. This represents an elongation of the molecule but no dimerization. P(R) functions of the scattering curves (Figure 1B) that were fitted to attain the experimental R_g_ both show a maximal intramolecular distance of 150 Å. The curve of apo RIG-I clearly flattens out around 100 nm, corresponding to the dimensions observed for various crystal structures while the curve of RNA-bound RIG-I accumulates larger distances between 100 nm and 150 nm, probably due to the release of the CARDs.

### RIG-I binding to synthetic RNA *in cellula* and activation of IFN-β promoter

**Figure 2:**
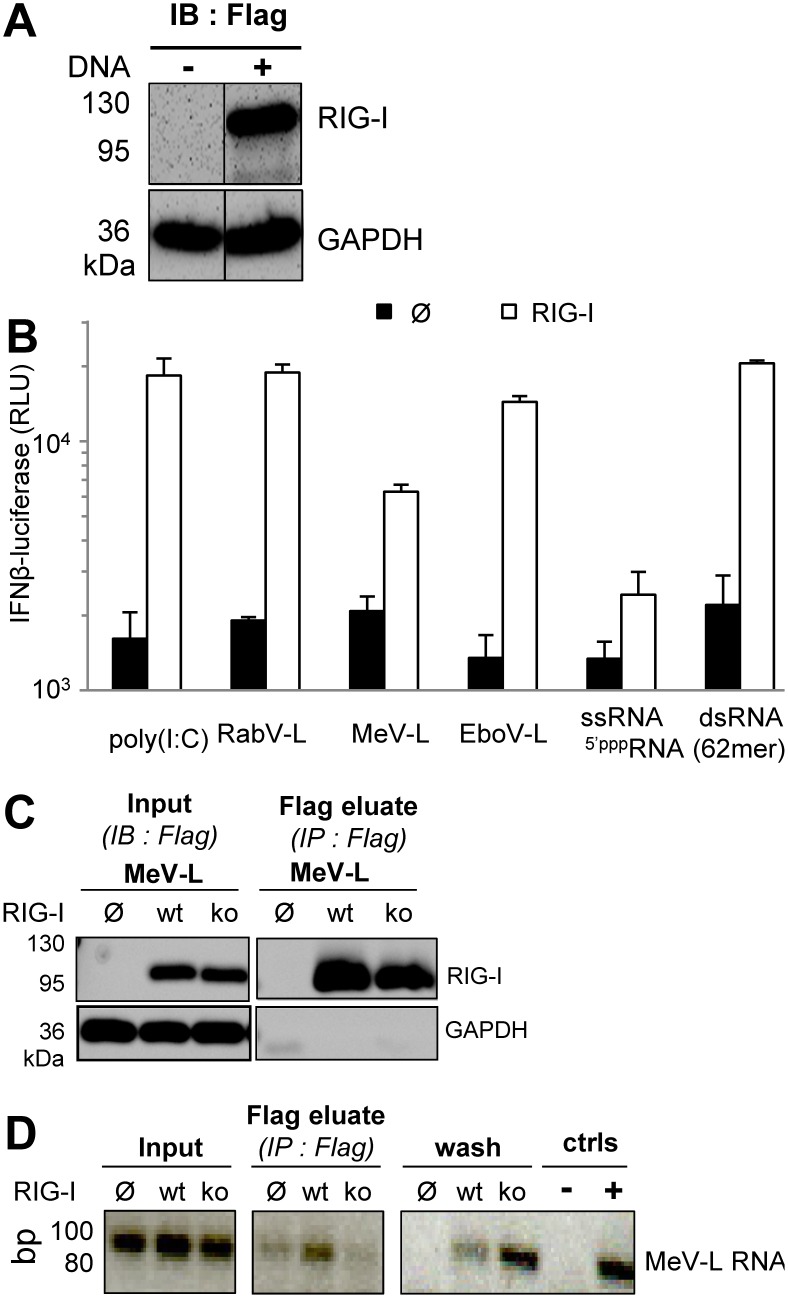
RIG-I binding to synthetic RNA and activation of IFN-β promoter. (A) Expression of Flag-RIG-I in Huh7.5 cells two days after transfection and analysed by western blot as revealed with Flag-specific antibody. (B) Luciferase expression driven under the control of the IFN-β promoter measured 24 h after transfection with 20 ng of synthetic RNA in Huh7.5 cells expressing or not Flag-RIG-I. (C, D) Immunoprecipitation of RIG-I:RNA complexes formed *in cellula*. Synthetic RNA were transfected in Huh7.5 cells previously transfected or not with Flag-RIG-I or Flag-RIG-I^ko^ 24 h before. Cells were harvested 6 hours after RNA transfection and RIG-I:RNA complexes were eluted from anti-Flag antibody immobilized on beads with a Flag peptide. (C) Specific immunoprecipitation of Flag-RIG-I as analysed by western blot. (D) RNA immunoprecipitated with Flag-RIG-I and analysed by RT-PCR.

Since RNA sensing by RIG-I results in IFN-β gene expression, the ability of the synthetic RNAs to induce the expression of a luciferase reporter gene under the control of the IFN-β promoter was tested. To avoid any interference of the endogenous innate immune response of the host cell, we selected Huh7.5 cells since they lack TLR3 and MDA5 expression, express a defective T55I RIG-I mutant and exhibit a poor feedback upregulation of RLR genes due to an IFNAR signalling defect [7], [35], [36], [37], [38]. In cells transiently expressing Flag-RIG-I (Figure 2A), the 62-mer-5′ppp-dsRNA was the best RIG-I stimulator, whereas the 62-mer-5′ppp-ssRNA induced only minimal luciferase activity (Figure 2B). While rabies leader (RabV-L) RNA was almost as good an activator of RIG-I as the 62-mer-5′ppp-dsRNA, the Ebola (EboV-L) and measles (MeV-L) leader RNAs induced intermediate and lower responses, respectively (Figure 2B). Rabies, Ebola and measles leader RNAs were T7-transcribed and purified by denaturing urea-PAGE. However, double-stranded side products cannot be totally avoided with this technique [9], [10], [39] and may explain their ability to activate RIG-I in the same way as the 62-mer-5ppp-dsRNA. It is also possible that these RNA could adopt different secondary structures enabling them to activate RIG-I. Alternatively, they can hybridize to cellular RNA since blasting their 5′ppp extremities revealed several >13 nt long complementary RNA transcribed sequences present in the human genome, although none have been identified as being enriched in RNA bound to RIG-I from measles virus infected cells [40].

Measles leader RNA was also tested for its ability to form *in cellula* complexes with RIG-I that are stable enough to be detected by immunoprecipitation of RIG-I. This synthetic RNA was transfected in Huh7.5 cells expressing wild type RIG-I or a RIG-I^ko^ construct associating a T55I mutation in the first CARD domain (inhibition of TRIM25 recruitment) with Q229A, T697A, E702A and K888/907A mutations in the helicase and CTD that prevent RNA binding to the corresponding domain [41], [42], [43]. Measles leader RNA was recovered in detectable amounts from eluted wt RIG-I, but not from its RNA-binding deficient RIG-I^ko^ counterpart (Figure 2C, D). This data is in agreement with the enrichment in leader RNA sequences found in complex with RIG-I from measles virus infected cells [40].

### Search for RNA induced RIG-I oligomerization *in cellula*

**Figure 3:**
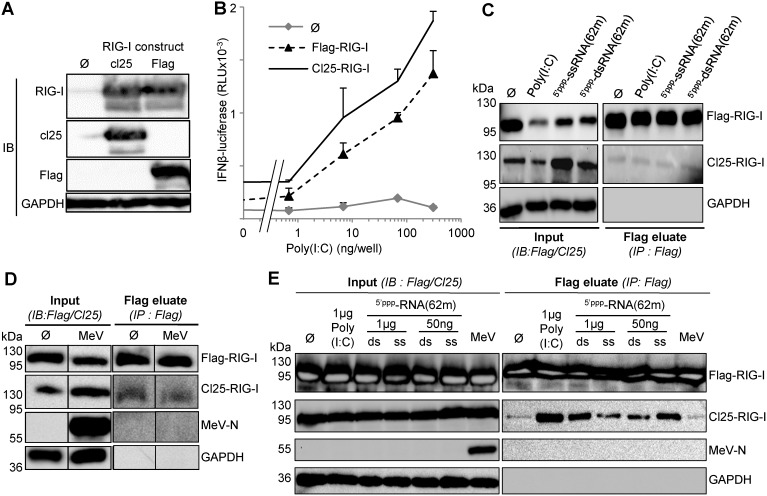
Analysis of RIG-I oligomerization *in cellula* as determined by co-immunoprecipitation 18 hours after stimulation by a cognate RNA ligand. (A) Similar expression of Flag-RIG-I and cl25-RIG-I constructs in 293T cells as revealed by western blot. (B) Efficiency of Flag-RIG-I and Cl25-RIG-I to activate the IFN-β promoter after Poly(I:C) transfection. See figure 2 legend for details. (C, D) Lack of co-immunoprecipitation of Cl25-RIG-I with Flag-RIG-I after their co-transfection in Huh7.5 cells and stimulation with Poly(I:C), ^5′ppp^ssRNA(62-mer) or ^5′ppp^dsRNA(62-mer) (C) or MeV infection (MOI 1) (D) as detected by western blot. (E) Nonsensical co-immunoprecipitation of Cl25-RIG-I with Flag-RIG-I expressed in 293T cells and after transfection of 1 µg or 50 ng of ^5′ppp^ds(or ss)RNA(62-mer) or MeV infection (MOI 0.5).

To determine whether RNA binding can induce RIG-I oligomerization *in cellula*, we built two expression vectors coding for RIG-I tagged with either Flag or Cl25 peptide. These constructs were expressed equally well as shown by similar signal in western blot revealed by anti-RIG-I antibodies (Figure 3 A). When expressed in Huh7.5 cells, both Flag-RIG-I and Cl25-RIG-I were stimulated by Poly(I:C) in a dose-dependent manner (Figure 3B) as expected from their strong expression levels (Figure 3A, C). To evaluate RIG-I oligomerization *in cellula*, Huh7.5 cells were co-transfected with Flag-RIG-I and Cl25-RIG-I constructs, stimulated by either Poly(I:C) or RNA transfection and finally harvested 18 hours after stimulation for an analysis in a co-immunoprecipitation assay. Cl25-RIG-I could not be co-immunoprecipitated with Flag-RIG-I, since similar trace amounts were detected in the absence or presence of RNA stimulation in Flag-RIG-I eluates (Figure 3C). Notably, Cl25-RIG-I could also not be co-immunoprecipitated with Flag-RIG-I after the transfection of the 62-mer-5′ppp-dsRNA although it has been shown to induce RIG-I dimerization *in vitro* (see Figure S3 in [30]).

Since we did not observe any RIG-I oligomerization *in cellula* after stimulation by Poly(I:C) or synthetic dsRNA, we tried to stimulate RIG-I by infecting cells with measles virus [42]. Huh7.5 cells were tested for their permissiveness to infection by Moraten-eGFP, a measles virus vaccine strain coding for eGFP as a viral reporter gene. Huh7.5 cells were infected by this virus as efficiently as were Vero cells that are commonly used for stock virus production (Figure S1 in File S1). We then tested the ability of measles virus infection to induce RIG-I oligomerization. Huh7.5 cells were co-transfected with Flag-RIG-I and Cl25-RIG-I, then infected or not with Moraten-eGFP virus and 18 hours later submitted to the immunoprecipitation assay. Both Flag-RIG-I and Cl25-RIG-I were strongly expressed (Figure 3D, inputs), but once again, we did not observe any detectable increase in the trace amounts of Cl25-RIG-I co-immunoprecipitated with Flag-RIG-I upon MeV infection (Figure 3D). We can exclude any pitfall in our procedures: the anti-Flag immunoprecipitation and elution procedure was well suited to detect the co-immunoprecipitation of N and P proteins from a recombinant MeV (Figure S2 in File S1) in agreement with the previously described interaction of these two proteins [44], [45], [46], [47], [48].

Since La Crosse virus (LACV) nucleocapsids that exhibited a triphosphorylated 5′ (5′ppp) terminus as does MeV can be co-immunoprecipitated with RIG-I from infected cells [49], we searched for any co-immunoprecipitation of the abundant MeV N protein with Flag-RIG-I, but none could be detected (Figure 3D). Incidentally, this observation confirms that 5-ppp (anti)genomic RNA from MeV cannot interact with RIG-I in physiological conditions likely because there are entirely covered by N protein as previously rationalized [42], [50].

RIG-I oligomerization was also tested in 293T cells stimulated by transfection of Poly(I:C), synthetic dsRNA or ssRNA, or Moraten-eGFP infection and 18 hours later submitted to the immunoprecipitation assay. Both Flag-RIG-I and Cl25-RIG-I were strongly expressed (Figure 3E, inputs). This time, detectable amounts of Cl25-RIG-I were found in the Flag-RIG-I eluate. However, this was observed whether Poly(I:C), 5′ppp-dsRNA or 5′ppp-ssRNA was co-transfected with RIG-I (Figure 3E), and independently of their ability to activate the IFN-β promoter (Figure 3B). Moreover, after transfection of only a 1/20^th^ amount of RNA, the amount of Cl25-RIG-I found in the anti-Flag immunoprecipitate decreased with the 62-mer-5′ppp-dsRNA but increased with the 62-mer-5′ppp-ssRNA. Reducing the amounts of transfected RNA and thus the number of RNA molecules accessible for one RIG-I would increase the chance for RIG-I to oligomerize. Since this assessment was only verified for the non-stimulatory ssRNA, we interpret the co-immunoprecipitation of Cl25-RIG-I with Flag-RIG-I as an experimental artefact likely due to over expression of RIG-I in 293T cells. All of these results were repeatedly observed using various experimental conditions, including the use of different lysis buffers.

### Search for early induced RIG-I oligomerization *in cellula*

**Figure 4:**
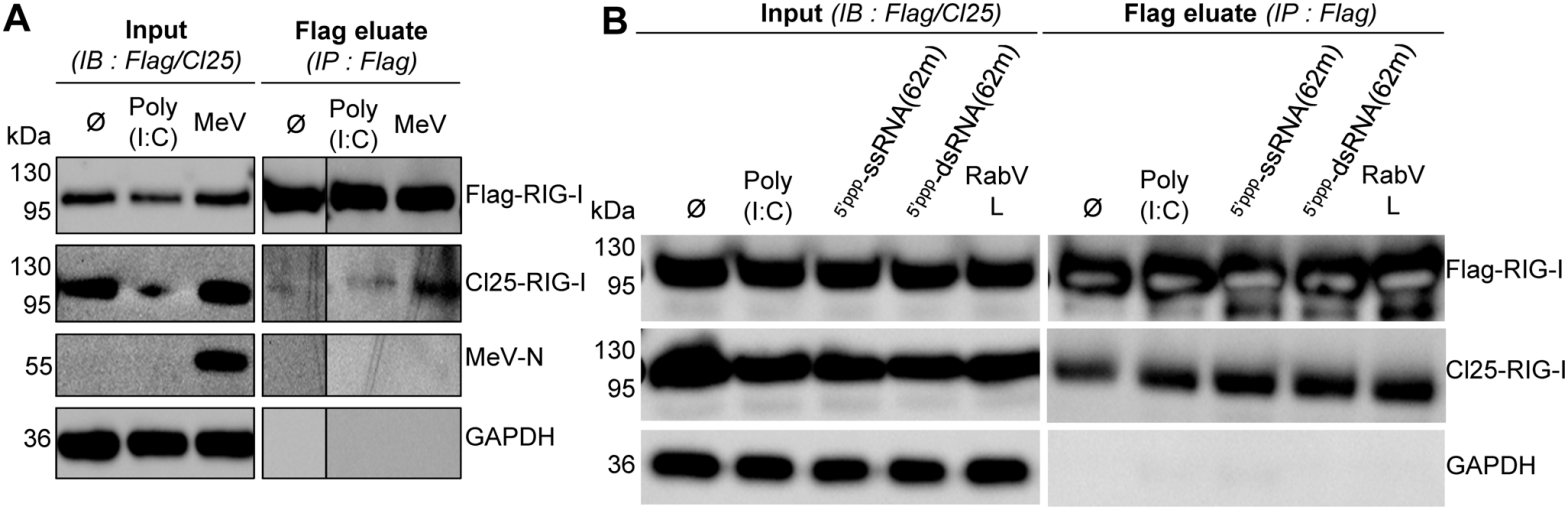
Analysis of RIG-I oligomerization in Huh7.5 (A) and 293T (B) cells determined by co-immunoprecipitation 4 hours after stimulation with Poly(I:C) (A, B), ^5′ppp^ssRNA(62-mer), ^5′ppp^dsRNA(62-mer) (B) or MeV infection (MOI 1, A, B) of cells expressing Flag-RIG-I and Cl25-RIG-I.

To verify the lack of cognate RNA-induced oligomerization of RIG-I *in cellula*, Huh7.5 cells were co-transfected with Flag-RIG-I and Cl25-RIG-I constructs, stimulated by either Poly(I:C) transfection or Moraten-eGFP infection and harvested 4 hours after stimulation for a co-immunoprecipitation assay. Both Flag-RIG-I and Cl25-RIG-I were expressed in Huh7.5 cells (Figure 4A, inputs). However, upon immunoprecipitation of Flag-RIG-I, Cl25-RIG-I again could not be clearly co-immunoprecipitated. Once more, MeV-N protein could not be detected in the immunoprecipitated fraction (Figure 4A). 293T cells were also used for the investigation of early induced RIG-I oligomerization. As for all experiments, Flag-RIG-I and Cl25-RIG-I were strongly expressed (Figure 4B, inputs), but in these conditions Cl25-RIG-I was evenly found in the Flag eluate independently of cognate RNA stimulation (Figure 4B).

### Search for RIG-I oligomerization *in cellula* using a protein complementation assay

**Figure 5:**
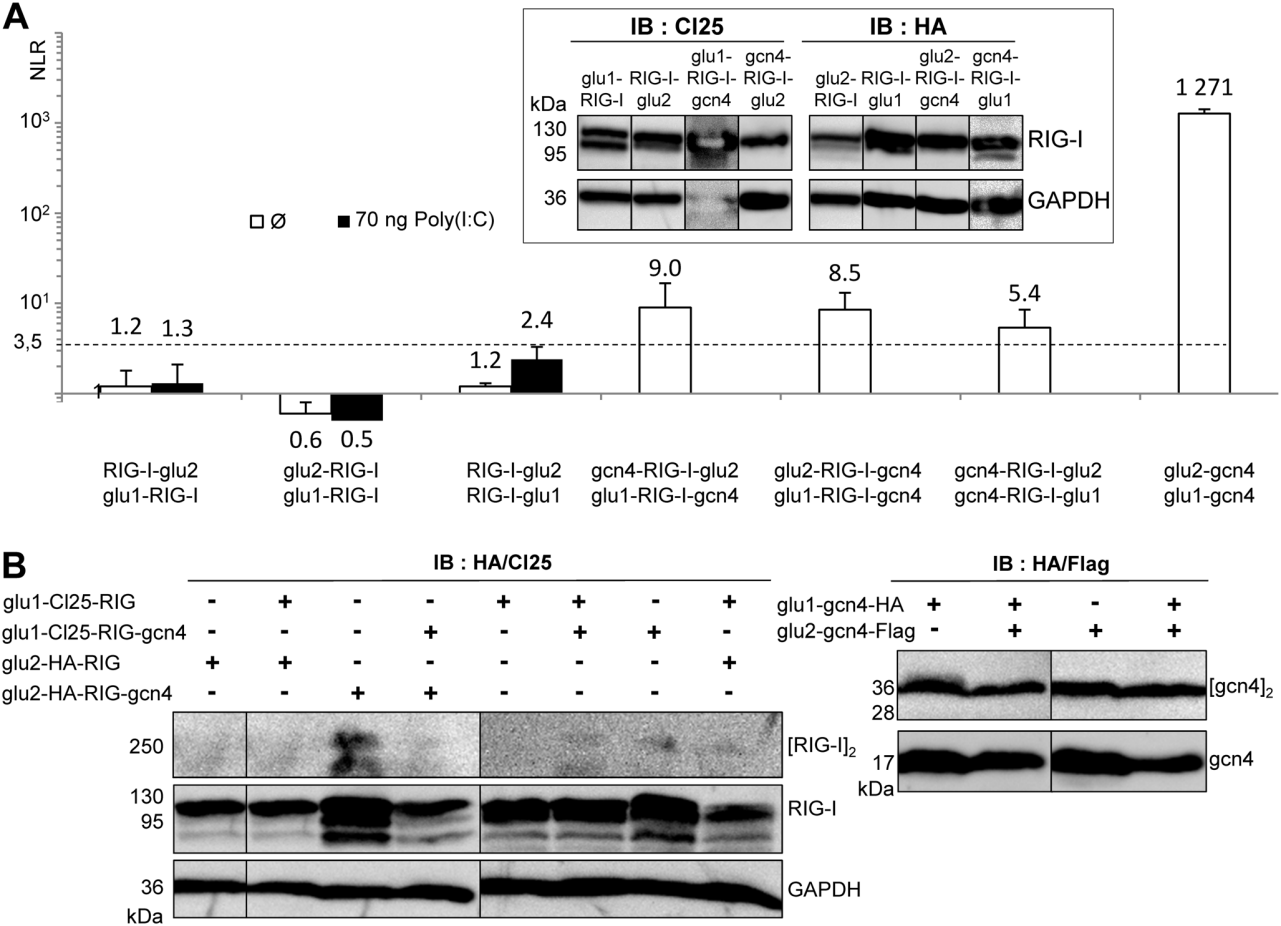
Lack of RNA induced RIG-I oligomerization *in cellula* as detected using PCA. (A) Ability of RIG-I/glu/gcn4 constructs to self-associate in the absence or presence of Poly(I:C) determined by PCA. Luciferase activity was measured 18 hours after transfection or not with Poly(I:C) in 293T cells transfected one day before with RIG-I/glu1/2/gcn4 constructs. (A, inset) Expression of chimeric RIG-I/glu1/2 constructs tagged with Cl25 or HA peptides in Huh7.5 cells two days after transfection as detected by western blot (note that the third sample (Glu1-RIG-I-GCN4 was overloaded, hence the overexposure of this protein and GAPDH). (B) Ability of RIG-I/glu/gcn4 chimeric proteins (left panel) and glu-gcn4 protein (right panel) for self-binding determined by western blot 24 hours post-transfection of 293T cells with glu1 or glu2 constructs alone or in combination. Lysates were separated without prior heat denaturation on SDS-PAGE before western blot analysis.

We reasoned that the co-immunoprecipitation assay might not be sensitive enough to detect RIG-I oligomerization induced by a cognate RNA. We therefore switched to the *Gaussia* Luciferase-Based Protein Complementation Assay (PCA). PCA has been described to be highly sensitive and have allowed us to detect interactions between monomers in the 0.2–1 µM range [33], [45], [51]. Cl25-RIG-I and HA-RIG-I coding sequences were fused at either the N- or C-terminus of *Gaussia* glu1 and glu2 split domains. All chimeric proteins were strongly expressed in Huh7.5 cells (Figure 5A). However, we did not detect any luciferase signal that would indicate basal or RNA-induced RIG-I oligomerization *in cellula* with any of the three tested combinations (RIG-I-glu2+glu1-RIG-I; glu2-RIG-I+glu1-RIG-I; RIG-I-glu2+RIG-I-glu1) (Figure 5A). It should be stressed that all glu/RIG-I constructs were able to be activated by a cognate RNA, indicating that grafting glu domains did not prevent RNA recognition by RIG-I. We then tried to force RIG-I dimerization by adding the leucine zipper gcn4 sequence to our constructs [26]. The glu1/2-RIG-I-gcn4 proteins were well expressed in Huh7.5 cells (Figure 5A). The addition of gcn4 sequence induced a modest and significant luciferase signal, whereas the co-transfection of glu2-gcn4 and glu1-gcn4 induces almost a 3 log higher signal (Figure 5A). Similar results were observed when the luciferase signal was measured only four hours after Poly(I:C) stimulation (Figure S3 in File S1). Moreover, the gcn4 sequence was accessible in the glu1/2-RIG-I-gcn4 chimeric proteins since they readily interacted with free gnc4 construct or gcn4 fused to another protein (Figure S4 in File S1). Correlatively, glu1/2-gcn4 dimerization was easily detected by western blot, while for RIG-I-GCN4 constructs, only a weak dimerization was detected when the glu2-HA-RIG-I-gcn4 protein was expressed alone (Figure 5B).

## Discussion

RIG-I oligomerization was proposed to occur during activation by a RNA ligand by two groups in 2007–2008 [14], [20]. Since then, the observation of RIG-I oligomerization has progressively become one of the landmarks of RIG-I activation, as many prominent papers in the field tend to report data supporting this idea [7], [10], [17], [18], [19], [21], [49], [52], [53], [54], [55]. However, the biochemical support remains rather poor, and the rationale enigmatic.

The RIG-I oligomerization concept originated from *in vitro* analysis by gel filtration of a mixture of pure RIG-I protein and short (from 19 bp to 135 bp) 5′ppp-RNA [10], [14], [53]. However, a significant shift of the volume of elution observed after chromatography does not necessarily indicate a linear augmentation of mass. Indeed the shape of the molecule can influence its migration properties through the reticulated gel and a conformational change occurs when RIG-I binds an agonist RNA with the tightening of the helicase around the RNA and the release of the CARDs [30], [56]. RIG-I oligomerization has also been observed by band shift in Blue Native Gel electrophoresis [20], [49]. In addition to some reliability concerns depending on the RNA source used to activate RIG-I [49], a band shift indicates a molecular change and does not necessarily prove oligomerization. The migration properties of a protein can be altered by a small bound RNA that is highly negatively charged and/or by its engagement into a multimolecular complex. In contrast, size-exclusion chromatography on a S200 column coupled to multi-angle laser light scattering analysis of mixtures of pure RIG-I protein with short dsRNA (see Figure S3 in [30] or synthetic *Mononegavirales* leader 5′ppp-RNA (this work) was compatible only with RNA/RIG-I 1∶1 monomer complexes. In agreement with our observations, RIG-I and hairpin duplexes of 10, 20 or 30 base pairs with a single 5′ppp end form 1∶1 complexes as analysed by analytical ultracentrifugation-sedimentation velocity [8]. Accordingly, crystal structures of RIG-I bound to short RNA (10 mers to 19 mers) shows only monomeric RIG-I:RNA complexes in a 1∶1 ratio [30], [53], [56]. Only when dsRNA contains two 5′ triphosphate ends, could RIG-I:RNA complexes be observed in a 2∶1 ratio [8], [30]. In these conditions, small angle X-ray scattering indicates that the RIG-I:RNA complex in the 2∶1 ratio adopts a very extended conformation [52]. The dimerization of RIG-I CTD reported previously [21], [57] may simply reflect the 5′ triphosphorylated bivalency of the dsRNA ligand used. Surprisingly, RIG-I dimerization in the presence of the 62-mer 5′ppp-dsRNA could not be observed *in cellula*. This could be explained by an unbalanced molar ratio of RIG-I protein to 5′ppp-dsRNA in the intracellular milieu, a competition with other 5′ppp-RNA binding proteins and/or the highly dynamic interaction of RIG-I with 5′ppp-dsRNA despite a K_d_ in the 160 pM range [58].

The incubation of very stable 5′ppp- panhandle RNA with dsRNA of variable length with cellular extracts from RIG-I transfected cells allows the observation of RIG-I oligomerization, at least if the dsRNA exceeds 46 bp in length [54]. According to the proposed model, one molecule of RIG-I would bind the RNA 5′ppp end and enter the RNA using ATP hydrolysis. Several RIG-I molecules would enter an RNA this way and form a RNA mediated oligomer. Contrary to the cooperative association of MDA5 along RNA, RIG-I molecules do not self-oligomerize to form a long filament but multiple proteins can bind to the same RNA, forming a RNA-poly-RIG-I scaffold that falls apart if the long RNA is cleaved by RNAse treatment [18], [54], [55].

*In vivo*, RIG-I oligomerization was reported once by pull down assay of Flag- and Myc-tagged RIG-I (see Figure 3 in [20]). However, the lack of clear differences between the data obtained in infected and non-infected cells, questions whether any RNA-induced RIG-I oligomerization had really occurred. In addition, multiple combinations of RIG-I and RIG-I domains and subdomains such as between RIG-I and CARDs, RIG-I and RIG-I-Δ-CARDs, CTD and CARDs, CTD and helicase, CTD and [helicase1+ helicase insertion domain] were also reported. While one cannot exclude that some of the reported interactions could reflect cis-interactions between RIG-I domains bridged or not by viral RNA (such as CTD/Helicase), the other interactions would suggest multiple oligomerization sites within RIG-I. However, none of them are supported by available RIG-I crystal structures. In contrast, in our work, we did not observe self-assembly of RIG-I upon recognition of synthetic or viral RNA by co-immunoprecipitation assay or using the more sensitive PCA assay. Furthermore, RIG-I dimerization hardly occurred even after being grafted with the gcn4 dimerization signal.

We strongly favour that a monomeric RIG-I-RNA complex is the minimal functional signal transduction unit in full agreement with biochemically defined monomeric RIG-I-RNA complexes that are able to activate the IFN response [8], [54]. Thus, so far there is no convincing evidence that, upon RNA recognition, RIG-I could (or should) self-oligomerize (i.e. via direct protein-protein interaction), and the model of RIG-I oligomerization for enabling signal transduction is inconsistent with all cell biological, biochemical and structural biological studies that have endeavoured to quantitatively assess the stoichiometry of RIG-I in its activated state. Rather, a single dsRNA can bind several RIG-I molecules and this can occur or not during viral infection [49] (and this work). Further down the signalling cascade, tandem CARDs of RIG-I associate with free K63 polyubiquitin in a helical tetramer complex [59] that becomes engaged in a complex interaction with membrane anchored MAVS. This scaffold would associate multiple RNA-RIG-I signal units to several MAVS molecules [12], [13], [53], [60], [61], [62], [63], [64]. Interestingly, this polyubiquitin-dependent scaffolding appears to be dispensable when several RIG-I molecules are associated with one long RNA [55] in agreement with RIG-I CARD tandem forming complexes with MAVS CARD [65].

## Supporting Information

File S1 Contains Figure S1, Efficient infection of Huh7.5 cells by Moraten-gfp MeV strain, at MOI 1. Vero cells and Huh7.5 cells were harvested 30 hours after infection and analyzed by flow cytometry for GFP expression with mean florescence intensity (left) and % of GFP expressing cells (right). Figure S2, The anti-Flag immunoprecipitation procedure can detect complex formation between MeV N and FLAG-P proteins. Vero cells were infected with two measles viruses expressing a wt P protein or a Flag-tagged P protein at MOI 0.1. The cell extracts, collected 20 h after infection, were immunoprecipitated with Flag antibody coupled to beads. Proteins eluted with Flag peptide were analyzed by western blot using anti-P 49.21 and anti-N Cl25 monoclonal antibodies. Note the exclusive pull-down of N from cells infected with the Flag-P virus. Figure S3, Ability of RIG-I/glu/gcn4 constructs for self-binding in absence or presence of Poly(I:C) determined by PCA. Luciferase activity was measured 4 hours after transfection or not with Poly(I:C) in 293T cells expressing RIG-I/glu1/2/gcn4 constructs. Figure S4, Accessibility of gcn4 sequence in RIG-I/glu/gcn4 constructs for determination of RIG-I oligomerization by PCA. Luciferase activity was measured 24 hours after transfection of RIG-I/glu1/2/gcn4, gcn4/glu1/2 and MeV Ntail/XD/glu1/2/gcn4 constructs in 293T cells.
(PDF)
