# Supplementary figures and images for: RIG-I Self-Oligomerization Is Either Dispensable or Very Transient for Signal Transduction
Source: PLoS One. 2014 Sep 26;9(9):e108770. doi: 10.1371/journal.pone.0108770 (PMC4178188; doi:10.1371/journal.pone.0108770)

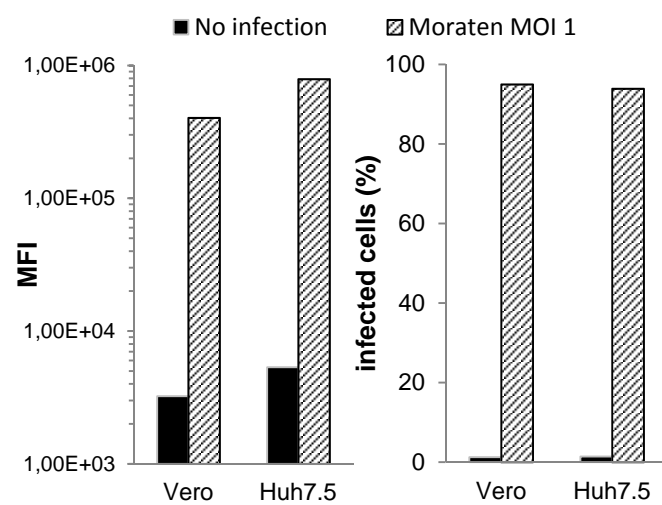

**Figure S1**

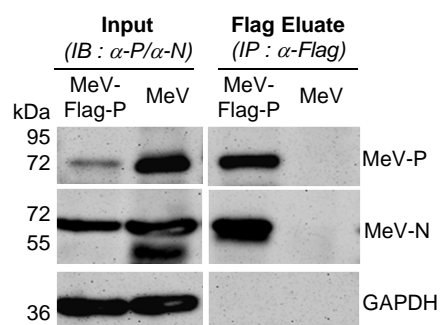

**Figure S2**

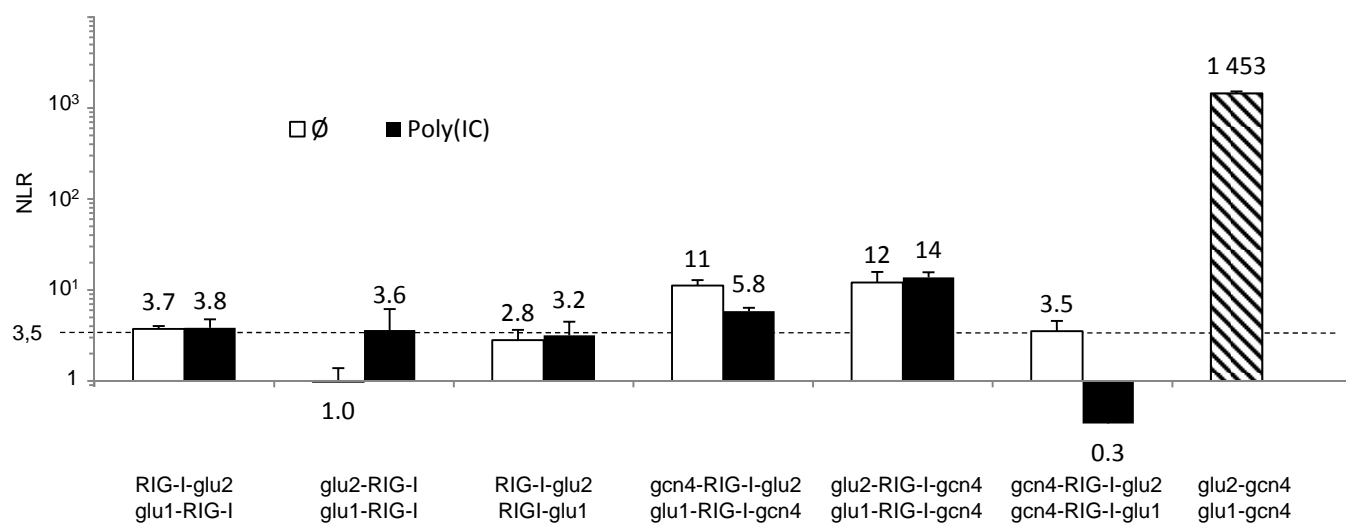

**Figure S3**

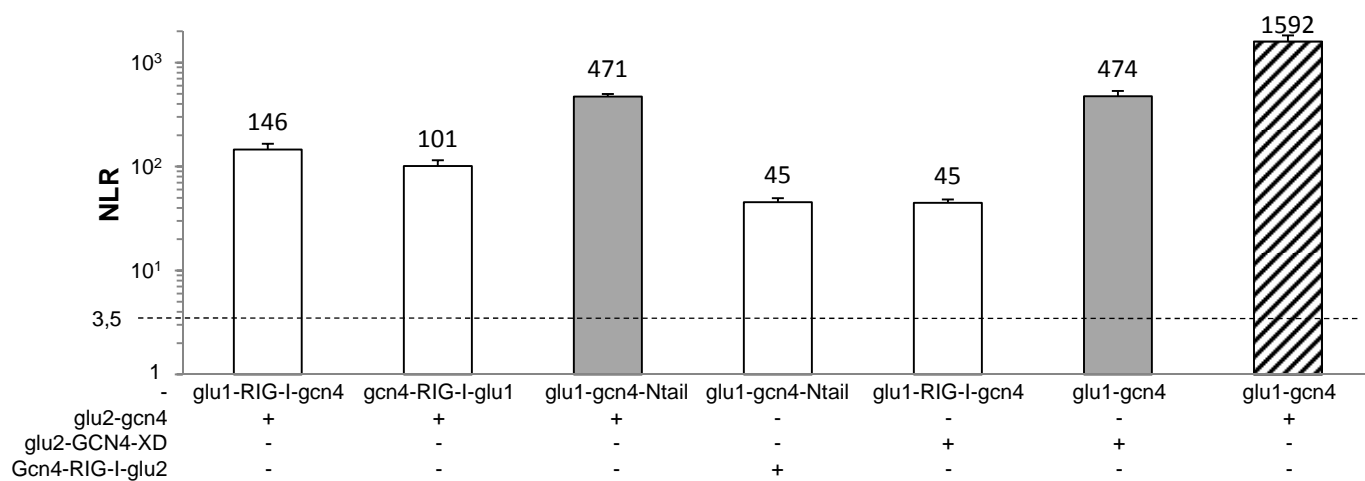

**Figure S4**

Supplement: File S1 — Contains Figure S1, Efficient infection of Huh7.5 cells by Moraten-gfp MeV strain, at MOI 1. Vero cells and Huh7.5 cells were harvested 30 hours after infection and analyzed by flow cytometry for GFP expression with mean florescence intensity (left) and % of GFP expressing cells (right). Figure S2, The anti-Flag immunoprecipitation procedure can detect complex formation between MeV N and FLAG-P proteins. Vero cells were infected with two measles viruses expressing a wt P protein or a Flag-tagged P protein at MOI 0.1. The cell extracts, collected 20 h after infection, were immunoprecipitated with Flag antibody coupled to beads. Proteins eluted with Flag peptide were analyzed by western blot using anti-P 49.21 and anti-N Cl25 monoclonal antibodies. Note the exclusive pull-down of N from cells infected with the Flag-P virus. Figure S3, Ability of RIG-I/glu/gcn4 constructs for self-binding in absence or presence of Poly(I:C) determined by PCA. Luciferase activity was measured 4 hours after transfection or not with Poly(I:C) in 293T cells expressing RIG-I/glu1/2/gcn4 constructs. Figure S4, Accessibility of gcn4 sequence in RIG-I/glu/gcn4 constructs for determination of RIG-I oligomerization by PCA. Luciferase activity was measured 24 hours after transfection of RIG-I/glu1/2/gcn4, gcn4/glu1/2 and MeV Ntail/XD/glu1/2/gcn4 constructs in 293T cells. (PDF) [file pone.0108770.s001.pdf]
